# A Three-Stage Teacher, Student Neural Networks and Sequential Feed Forward Selection-Based Feature Selection Approach for the Classification of Autism Spectrum Disorder

**DOI:** 10.3390/brainsci10100754

**Published:** 2020-10-19

**Authors:** Naseer Ahmed Khan, Samer Abdulateef Waheeb, Atif Riaz, Xuequn Shang

**Affiliations:** 1 School of Computer Science and Technology, Northwestern Polytechnical University, Xi’an 710072, China; naseerkhan@mail.nwpu.edu.cn (N.A.K.); samirabdulateef@mail.nwpu.edu.cn (S.A.W.); 2 Department of Computer Science, University of London, London WC1E 7HU, UK; atif.riaz@city.ac.uk

**Keywords:** ABIDE, autism spectrum disorder, classification, connectivity matrix, feature selection, fMRI, rs-fMRI

## Abstract

Autism disorder, generally known as Autism Spectrum Disorder (ASD) is a brain disorder characterized by lack of communication skills, social aloofness and repetitions in the actions in the patients, which is affecting millions of the people across the globe. Accurate identification of autistic patients is considered a challenging task in the domain of brain disorder science. To address this problem, we have proposed a three-stage feature selection approach for the classification of ASD on the preprocessed Autism Brain Imaging Data Exchange (ABIDE) rs-fMRI Dataset. In the first stage, a large neural network which we call a “Teacher ” was trained on the correlation-based connectivity matrix to learn the latent representation of the input. In the second stage an autoencoder which we call a “Student” autoencoder was given the task to learn those trained “Teacher” embeddings using the connectivity matrix input. Lastly, an SFFS-based algorithm was employed to select the subset of most discriminating features between the autistic and healthy controls. On the combined site data across 17 sites, we achieved the maximum 10-fold accuracy of 82% and for the individual site-wise data, based on 5-fold accuracy, our results outperformed other state of the art methods in 13 out of the total 17 site-wise comparisons.

## 1. Introduction

The human brain is considered the most complex organ in the body due to it’s structural and functional variations across the temporal and spatial domain, variety of cognitive functions based on the interaction of functional regions and the intrinsic modularity that is present in those regions [1]. Although, there are influential studies that have shown the existence of small-world-networks [2], modular networks [3] and hierarchical organization of the different modules [4] in the human brain but still much needs to be done in the field of brain sciences to grasp the complexity of the brain. New theories and understanding of the coordination and synchronization of various brain regions are being explored to solve the mysteries of the functions of the brain networks [5].

Autism Spectrum Disorder (ASD) is a brain disorder with characteristics such as social aloofness, repetitive actions and inability to communicate effectively. Although, there are many neuroscience and genetic related markers identified already but still a lot of progress needs to be done for the diagnosis of this mental disorder [6].The prevalence rate of parents reporting autism in children in the USA between the age of 3 years to 17 years, using a sample size of more than 78,000, was estimated to be 110 per 10,000 [7]. Gender is also believed to be a factor when it comes to autism disorder and studies have proved that the autism disorder is more common in men as compared to women as discussed in [8] and [9]. More recent studies have also proved that the gender factor has significance in ASD and cannot be taken as a trivial issue. In this regard meta-analysis of 54 studies with a combined size population of 13,784,284 discovered that among the 53,712 participants that had ASD, male population of 43,972 and female of 9740 were found to have ASD resulting in 3:1 prevalence in the males as compared to females, thereby, providing strong evidence that autism more likely affects men [10] and verifying an earlier work which found that autism was more common among men [11].

Many imaging modalities are used to study ASD and each of these modalities have their own peculiar characteristics. These modalities include DTI (Diffusion Tensor Imaging), EEG (Electroencephalography), fMRI (functional Magnetic Resonance Imaging), MEG (Magnetoencephalography), PET (Positron Emission Tomography) and SPECT (Single-Photon Emission Computed Tomography) are all used in practice for the study of autism [12,13,14,15,16].

Functional Magnetic Resonance Imaging (fMRI) is currently very popular as it is a non-invasive method to acquire neural activity information from the human brain. Moreover, recent advancements in the fMRI technology has made considerable improvements in the quality and reliability of the haemodynamic BOLD (Blood-Oxygen-Level-Dependent) signal at both the spatial as well as the temporal level that is obtained from the powerful magnetic scanner [17,18]. The primary task of an fMRI experiment which is based on the MRI (Magnetic Resonance Imaging) is to measure the signal using 3D scans of the brain which are stacked in the time dimension that makes them a 4D modality. After that, brain volumes are divided into small cubes called “voxels” and then the average BOLD signal or neural activity in those voxels is measured [19].

In many brain disorders, functional connectivity based analysis is getting more and more attention due to its simplicity in calculation and understanding as it is a measure that tells how close functionally related two brain regions are with each other. Studies have shown alteration patterns in the functional connectivity and existence of significant and interpretable markers from the brain region wise connectivity matrix that are discriminating between a subject of the disease and a healthy control in a variety of brain disorders such as ADHD (Attention Deficit Hyper Disorder), Alzheimer, ASD, Epilepsy and Schizophrenia [20,21,22,23,24].

In this study, we have proposed a deep learning-based Teacher-Student feature selection method to extract the most discriminating features between the autistic subjects and the healthy controls. Firstly, we built the functional connectivity matrices for each subject in the dataset, as this would be the input to our neural network-based models. Secondly, we constructed a large neural network which we call a “Teacher” neural network and trained it on the connectivity matrix so that it could learn insightful patterns on the training dataset. Thirdly, we extracted the codes from the trained Teacher neural network and normalized them for convergence, these codes can be called as a learned embedding on the trained Teacher model. In the next step, we built a one layer hidden neural network, which we call as “Student” neural network and trained it again on the connectivity matrix input and the task of this neural network was to reproduce the codes or embeddings that we extracted from the trained teacher neural network. After this, we extracted the weights from the hidden layer of the Student neural network and sorted them in descending order as higher magnitude of the weights correspond to the indices of the connectivity matrix features with greatest discrimination power. Finally, we gave those features to various classifiers and tested the sensitivity, specificity and accuracy of the classifier. Our contribution in this study has three key characteristics, (i) first we used the 3-Stage Teacher, Student and SFFS based feature selection approach, which is a novel idea for the fMRI domain, (ii) our features are of a short size and are verifiable from the supplementary material which is mentioned in our study and lastly we have outperformed several state of the art algorithms in the literature which validates the effectiveness and usefulness of our work.

The rest of the paper is as follows: in Section 2 we will discuss the related research that has been conducted on the current problem; in Section 3 will discuss dataset and the proposed methodology; in Section 4, we will discuss our experiments, results and comparison with the state of the art methods; in Section 5 we will discuss our feature selection process; in Section 6 we will discuss our selected features and their significance in the autism disorder, and lastly in Section 7 we conclude our study and propose future work for the research community on this problem.

## 2. Related Research Work

ASD has grabbed the attention of many researchers due to the challenging task of finding discriminating neural markers that could help differentiate an autistic person from a healthy control. In the following subsections, we will discuss various techniques that are being used for the study of classification related to fMRI imaging dataset available on ASD.

### 2.1. Signal Processing Based Approaches

A study explored the Generalized Autoregressive Conditional Hetroscedasiticty model (GARCH) [25] that extracted features after decomposing the subject region data into sub-bands using Double Density Dual-Tree Discrete Wavelet Transform (D3TDWT). After that, the extracted features were given to Support Vector Machine (SVM) that resulted in 71% accuracy for male and 83% accuracy for female ASD data. Fast-Entropy based algorithm [26] was used and the results were reported using the area under the receiver (AUC) metric value of 0.62 for the classification of ASD, Discriminating features on 21 autistic and 26 healthy patients were selected using two-sample t-test and were fed to SVM classifier for training. In [27] authors applied fluctuation entropy using a single functional near-infrared spectroscopy channel on 25 ASD and 21 control subjects resulted in that 97.8% accuracy. A variation of Fourier Transform (FT) called Graph Fourier Transform (GFT) was applied on 172 subjects and control data where the author first computed the statistical measures from the healthy time series of a subject then projected that to a structural graph which was computed from the healthy connectome graph that resulted in better classification. In another study [28] using ICA (Independent Component Analysis) and their associated time series, wavelet-based coherence maps were built which when given to classifier for training, resulted in 86.7% and 80% testing accuracies on two different datasets containing 12 ASD, 18 controls and 12 ASD and 12 controls respectively. In [29] authors proposed a spatial filter approach that projected the covariance matrix of the BOLD signal of the ASD and control subjects to the orthogonal directions to make the two type of subjects highly separable.

### 2.2. Functional Connectivity Based Approaches

Functional Connectivity (FC), a pair-wise relationship between two brain regions, is considered an important step in the search for the neuromarkers for the ASD subject. A study applied ICA to study altered FC in the brain Default Mode Network (DMN) on the 16 ASD patients with matched 16 control subjects showed promising biomarkers for the classification of ASD subjects [30], as their results showed a decreased FC among the ASD subjects as compared to control subjects. Disruption in FC was found in task unrelated neural activity between the 23 ASD and 20 control subjects, 17 Regions of Interest (ROI) were used to form a 17×17 correlation matrix that was used to study the altered FC by using two sample t-test between the two connectivity matrices [31]. In [32] authors showed that altered FC is important for discriminating ASD subjects from the healthy control after controlling the confounding effect of head motion, their findings concluded altered FC in 19 ASD and 20 control subjects. In another study a Probabilistic Neural Network (PNN) [33] consisting of an input layer, pattern layer, summation layer and output layer showed promising results on the large data consisting of 312 ASD and 328 control subjects. The authors used a correlation-based connectivity matrix in the input layer resulting in 90% test accuracy. In another study, FC was also found to be predicting the future traits of ASD based on three brain networks involving Default Model Network (DMN), Fronto Parietal Task Control Network (FPTN) and Salience Network (SN) but their results showed promising results only in SN with 100% sensitivity and 70% precision [34]. In [35] authors analyzed the aberrant connections in the FC correlation matrix on the ROI made from the selected ICA components. They constructed the connectivity matrix of size 54×54 for each type of subject and clustered those connectivity matrices into a different number of groups using the varying value of the parameter k in the k-means clustering algorithm. Finally, a two-sample t-test was performed on each of the Mean Dwell Time (MDT) for all k values to extract significant regions between ASD and control. In [36], the authors incorporated both intra-site and inter-site variability to validate their results. They used multiple brain atlases to first estimate the time-series of the ROIs and then constructed the features from the estimated connectivity matrix. Features were given to the ridge-classifies resulting in 67% accuracy on the 1112 subjects data compiled from multiple sites. Adapted Signal Change (ASC) [37] approach was introduced to improve the specificity of results obtained using the connectivity matrix-based analysis. Authors showed that alterations in the time series signal can be decomposed into the prevalent classes of change that are more useful in the subsequent analysis done using connectivity matrices. Support Vector Machine-based Recursive Feature Elimination (SVM-RFE) was proposed by [38], where the correlation-based connectivity matrix was recursively pruned for discriminating features using SVM classifier, resulting in 90% accuracy on the dataset combined from all sites. Eigen features corresponding to 256 brain regions using the Laplacian matrix were proposed in [39] with the accuracy of 77%.

### 2.3. Deep Learning-Based Approaches

Deep learning [40] is based on the architecture of neural network where neurons are attached in a layer-wise fashion called feed-forward connection with a non-linear activation at the final processing unit on each layer and training of each layer based on the back-propagation algorithm. In this way, a complex relationship is modelled and a representation or latent dimension of the input model is attained that could better explain the underlying relationship. Long Short-Term Memory (LSTM)-based classification of the ASD dataset was proposed in [41]. In the first step, data augmentation was first performed based on the minimum time length of 90. Instead of calculating the connectivity matrix and then feature selection, the authors directly classified the subjects based on the individual times series resulting in 68% accuracy on the combined dataset. Deep Neural Network-based Feature Selection (DNN-FS) was proposed in [42]. First, the connectivity matrix features were fed to the sparse autoencoders to reduce the dimensionality of the dataset. After this step, data were transformed to a lower-dimensional representation. Finally, it was fine-tuned by attaching a neural network thereby extracting the features with high discriminating power resulting in 86% classification accuracy. Phenotypic information on the ASD and control data has also found to be effective in discriminating ASD subjects from the control. Phenotypic data is concatenated with the features data for the input of LSTM network resulting in 67% accuracy [43]. Layer wise pre-trained autoencoders were used in [44], two autoencoders of nodes 1000 and 600 were separately trained on the input features consisting of 19900 nodes (CC200 brain atlas) and the 1000 nodes respectively. Then they were fine-tuned by attaching a classification layer consisting of two nodes resulting in an overall accuracy of 70% after cross-validation. Deep Belief Network (DBN) was used to classify the ASD subjects from the healthy controls, both structural fMRI and resting-state fMRI were used to extract the features that were fed to stacked Restricted Boltzmann Machine (RBN)-based DBN. It resulted in 65% accuracy in the discriminating classification of ASD and healthy controls [45]. A Convolution Neural Network (CNN) consisting of two sub-networks, that is one CNN to extract the time-related components from the time series data and the other to extract the spatial features from the data using a 3D CNN was proposed in [46] resulting in F-score of 89%. A two-stage Deep Neural Network (DNN) classifier was proposed in [47]. First, a CNN-based model was built to extract important features from the data then images were corrupted using frequency normalized approach and finally, the extracted features were again given to DNN that was used to get the discriminating features from the ROIs. Auto-ASD-Network was proposed in [48] to classify the ASD subjects from the healthy control data. Firstly, the dataset was augmented using the Synthetic Minority Oversampling Technique (SMOTE) and a neural network was built for classification. Finally, the hidden layer of the neural network was connected to the ATM (Auto Tune Model)-based SVM to classify the data. ASD-Diagnet was proposed in [49], first, the data augmentation was performed using a linear interpolation method. Then a single layer perceptron and an autoencoder was used to classify the data on 1035 participants that resulted in 80% overall accuracy. A CNN based deep neural network and a Multichannel based attentional neural network for the classification of ASD were proposed in [50] and [51] respectively with promising results.

## 3. Materials and Methods

### 3.1. Dataset

We have used ASD preprocessed dataset from the Autism Brain Imaging Data Exchange (ABIDE) [52] consortium where data from 17 sites with phenotypic information related to age, sex and Autism Diagnostic Observation Schedule (ADOS) [53] scores are maintained in a pre-preprocessed state for researchers. It is pertinent to mention here that resting-state rfMRI imaging data is computationally expensive to download, store and process. The original ABIDE dataset claimed to have 1112 subjects consisting of 539 ASD and 573 Healthy controls subjects. However, after removing subjects with missing information we have a total of 1035 subjects which is consistent with the total subjects reported in earlier studies of [37,44]. A dataset with the total number of participant’s condition in each site is summarized in Table 1.

### 3.2. Preprocessing the Dataset

We have used the ABIDE dataset that is pre-processed using Configuration Pipeline for the Analysis of Connectome (CPAC) [54], CPAC consists of open source tools that involve both structural and functional processing of the rsfMRI-based imaging data. Where the structural preprocessing involves steps like skull stripping, segmenting brain into tissue types and normalizing them to Montreal Neurological Institute-based template. Whereas, the functional processing includes steps like slice time correction, motion correction, band-pass filtering and registering the functional images to anatomical space.

### 3.3. Methodology

**Figure 1:**
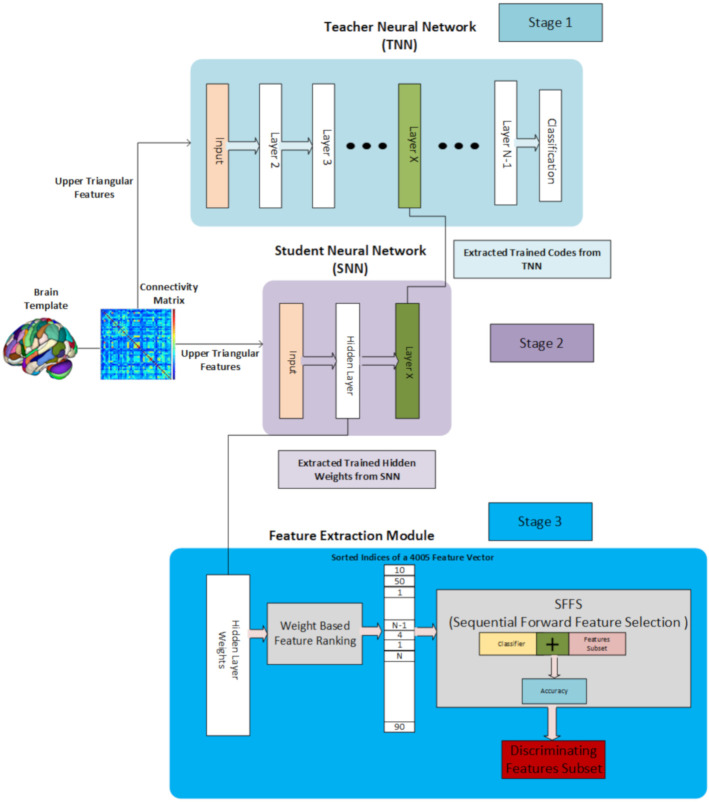
3 Stage Teacher-Student-based and SFFS-based Feature Selection Architecture.

Our feature selection approach is inspired by the deep learning-based feature selection method proposed in [55], in which they developed a knowledge distillation-based deep learning approach to extract the features from the dataset. Our proposed approach consists of 3 stages for the Feature Selection process that is shown in Figure 1.

#### 3.3.1. Stage 1, Teacher Neural Network

In the Stage 1, a Teacher Neural Network (TNN) which is a large neural network that consists an input layer, a number of hidden layers which is much larger than the Student Neural Network (SNN) and a binary classification layer. The dimension of the input layer is the same as the dimension of the feature vector and neurons in each subsequent layers are gradually reduced to avoid over-fitting. Finally, the last layer is a classification layer as our dataset is binary class data with autistic subjects labels set to 1 and healthy controls set to 0, so the last layer contains two neurons for each of the mentioned class.

#### 3.3.2. Stage 2, Student Neural Network

In the second stage, After Training the TNN we extracted the trained codes of the hidden layer of the TNN and gave Student Neural Network (SNN) a task of reproducing these codes as in the case of simple autoencoder-based neural network model. We used a lower dimension for the embedding layer as the higher the size of the embedding layer, the more parameters it will contain in the SNN resulting in poor training and higher loss. It is pertinent to mention here that the SNN just contains one hidden layer with customized regularity conditions on the weights of the hidden layer that is explained in [55]. After training the SNN we extracted the weight matrix corresponding to the input feature layer and hidden layer as our objective is to select features based on this weight matrix.

#### 3.3.3. Stage 3, Feature Extraction Module

In the last stage, Feature extraction is on the hidden layer’s weights that are extracted from the trained SNN. Selection of discriminating and meaningful features is done, by first, sorting the trained weights in order of increasing magnitude. Secondly, the feature indices from the input feature vector are extracted using the corresponding ranked weight vector. Lastly, a Sequential Forward Feature Selection (SFFS) approach is used to select a set of the most discriminating features in a linear stepwise way, that is we started from one feature and stopped the feature selection when the maximum accuracy got attained on the trained data using a selected classifier.

#### 3.3.4. Algorithm

Consider that $S_{i}$, i=1,2,…N is a subject matrix denoting *i*th autistic or healthy subject’s information where *N* denotes the number of subjects. Moreover, each $S_{i}=X_{j}^{k}$ where $X_{j}^{k}$ denotes the time series data for each subject such that j=1,2,…R and k=1,2,…T, where *j* corresponds to the brain region out of *R* total regions and *k* denotes the time point out of the *T* total time points for each subject. Our algorithm’s input is the subject connectivity matrix which is obtained by calculating a pairwise correlation between two brain regions. Therefore, first, we need to convert the brain region time series data from each subject to the region-wise correlation matrix that is, $Ci=Correlation(S_{i})$ where $C_{i}$ is a R×R matrix, computed by taking Pearson correlation of each brain region time series with the rest of the regions resulting in a symmetric square connectivity matrix for each subject. $F_{i}=UpperTrianguler\left(C_{i}\right)$ is a feature vector that contains the upper-triangular elements of the connectivity matrix as only the unique elements of this matrix will be useful for further analysis. The number of elements in the feature vector are of size $\frac{R(R-1)}{2}$ where *R* depends upon the selected brain parcellation template like AAL, CC200 and dosenbach160. Details of the working of our 3-Stage Feature selection method that converts the connectivity features $F_{i}$ to the indices vector and the SFFS method that on getting $F_{i}$, index vector, a specified threshold accuracy level and a classifier, sequentially selects the features are described in Algorithm 1 and Algorithm 2, respectively.
**Algorithm 1** Ranking Discriminating Features Algorithm**Input: $CONN_{m\times n}$** **Output: $Indices_{S\times 1}$**1:$CODES_{m\times d}\leftarrow TeacherNeuralNetwork\left(\mathrm{CONN}\right)$2:$W_{m\times h}\leftarrow StudentNeuralNetwork\left(\mathrm{CONN},\mathrm{CODES}\right)$3:$Indices_{S\times 1}\leftarrow Diag\left(W.W^{T}\right)$4:$Indices_{S\times 1}\leftarrow ArgSort\left(Indices\right)$

**Algorithm 2** Sequential Forward Feature Selection Algorithm
**Input: $CONN_{m\times n}$**, $Indices_{L\times 1}$, THR,CLF

**Output: $F_{S\times 1}$**

1:
featureBox←empty
2:
$ACC_{L\times 1}\leftarrow 0$
3:**for**indexin *Indices***do**4: features←*CONN*[0to  index]5: ACC←AccuracyScore(CLF[features])6: **if**ACC is = THR **then**7:  featureBox.append(*features*)8: 
**end if**
9:
**end for**
10:maxIndex←ArgMax(*ACC*)11:F←(*featureBox[maxIndex]*)


## 4. Experimentation and Results

We have done our experiments on both the combined dataset and on the site-wise data to prove the effectiveness of the selected features based on our 3-Stage-based feature selection approach.

### Experimental Settings

We have used the brain Automated anatomical labelling (AAL) template which divides the brain into 90 regions. Specific details about the AAL regions see Table S5 in Supplementary Materials, the reason for choosing the AAL template is the fewer number of correlation features, which are 4005 unique paired regions, thereby, avoiding the curse of dimensionality as explained in another work on the ADHD brain disorder classification [56]. For each autistic subject and healthy control, we first calculated the functional connectivity between the brain regions. The FC is of size 90×90 correlation matrix where the correlation is computed using Equation (1), where $x_{i}$ and $y_{i}$ correspond to the time series region values corresponding to region *x* and *y*, $\bar{x_{i}}$ and $\bar{y_{i}}$ correspond to the average values against those regions and lastly, *n* is the total number of time points.

(1) $$corr\left(x_{i},y_{i}\right)=\frac{\sum_{i=1}^{n}\left(x_{i}-\bar{x_{i}}\right)\left(y_{i}-\bar{y_{i}}\right)}{\sqrt{\sum_{i=1}^{n}{\left(x_{i}-\bar{x_{i}}\right)}^{2}\sum_{i=1}^{n}{\left(y_{i}-\bar{y_{i}}\right)}^{2}}}$$

For TNN we have made a relatively large neural network as compared to the SNN, Our TNN is a 17 layer neural network including the input and output layer, neurons in each layer are gradually reduced from the original 4005 input dimension to the final binary layer corresponding to autistic and healthy controls. TNN is trained with “Adadelta” optimizer with the learning rate set to “0.001” and the loss is set to ”binary-cross-entropy”. After training the TNN with the defined parameters, we extracted the trained TNN until the layer of size “5” and the complete dataset was transformed to the latent dimension of size 5 instead of the original 4005 input dimension, for specific details about the architecture and parameters of the TNN please see the Tables S1 and S2 in the Supplementary Materials. The justification for choosing the embedding layer of size 5 is that we did experiments with embedding layer of size greater than 5 and size lesser than 5. For the larger embedding dimension, the overall accuracy was not good which led to poor results in site-wise accuracy as well. Similarly, a lower dimension embedding size gave lesser accurate results in site-wise accuracy and could not learn the complexity of the dataset. All those experiments proved that embedding size of 5 was optimum for our study. SNN is a shorter neural network with only one hidden layer of size 100, where the size of 100 in the hidden is chosen after doing experiments with other sizes, the purpose of the SNN is to learn the embeddings of size “5”. Therefore, the SNN input is the 4005 input dimension, 100 in the hidden layer and the final layer is of size “5”. Training of the TNN is done by setting the optimizer to “Adadelta”, learning rate of “0.01” and loss function set to “mse”. We now give the justification for choosing the specific hyper-parameters values that we used after experiments. The number of hidden layers of the neural network is still a challenging problem that needs much attention. We used a 17 layer neural network for the TNN after experiments with 10-layer, 15-layer and 20-layer neural networks as possibilities for choosing a specific number of neural network’ layers are just endless. The gradual decrease in the number of neurons for the TNN and in the dropout is to avoid over-fitting, as the large dropout value ignored many useful features. The lower learning rate of 0.001 in the TNN was used as the higher learning rate resulted in much larger variations in the loss values during training. The number of epochs was chosen as 500 with a batch size of 16 as lower learning rate converges at a slower speed and a smaller batch will not ignore too many subjects or examples. The binary-cross entropy loss function in the TNN and mean squared error loss function in the SNN is used as the former is the classification task and the latter is the reproduction task. The optimizers “Adadelta” and “Adam” did not have any difference in the results, so to make our experiments consistent, we used the same optimizer in TNN and SNN. A comparatively higher learning rate of 0.01 was used in the SNN as it is one layer network with the task of reproducing the embedding values and with thousands of parameters in the first two layers so it’s loss values were converging very slow which prompted us to increase the learning rate. The activation function of “tanh” was used in the TNN as the dataset contains negative values so using the “relu” activation resulted in the loss of information and resulted in poor convergence during training. On the other hand activation function of “relu” was used in the SNN as the embedding layer was first standardized for convergence during the training of SNN. Lastly, for the training of SNN whole dataset was used because just using a feature set of size 4005 will lead to over-fitting and we wanted the latent lower dimension representation of the whole dataset. Specific details about the architecture and parameters of the SNN, please see Tables S3 and S4 in the Supplementary Materials. Both the TNN and SNN get the input in the form of correlation matrix-based connectivity features, where the number of layers is chosen after doing experiments.

## 5. Feature Selection

**Figure 2:**
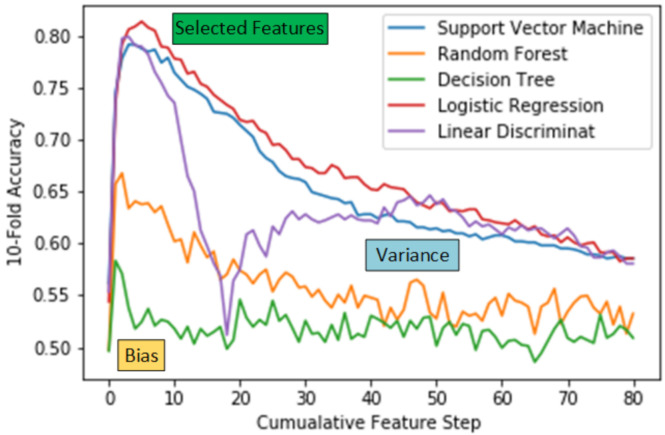
Comparison of Cumulative Feature Selection on the 10-Fold Accuracy.

We have employed the sequential forward feature selection (SFFS) approach to get the most discriminant features that differentiate an autistic subject from the healthy control. In forward feature selection, features are appended to the training vector in a forward direction and are fed to the classifier to measure the accuracy. We have tested our feature selection approach using five different classifiers, which are Support Vector Machine (SVM), Random Forests, Decision Trees, Logistic Regression-based classifier and Linear Discriminant classifier. After obtaining the indices of the most discriminant features using the trained SNN, we selected a sequential group of these indices to form a set of features that were fed to the classifier. We have evaluated the method using 10-fold Accuracy using various classifiers with the cumulative feature step of 50 features at each forward step, by cumulative feature step of 50 we mean that, each step of 1 means a set of 50 features, for example, step of 1 means features in range 1 to 50, step of 2 means features in the range 1 to 100. Here it is pertinent to mention that step larger than 1 is used to speed the classifier training process. 10-Fold accuracy of various classifiers with the cumulative step as defined above is shown in Figure 2. It can be seen from the figure that accuracy of the classifier is highest in cumulative step 1 to 5 and after that, it starts to decrease, which is a typical case of over-fitting as the selection of large features means “curse of dimensionality”. Selected cumulative set with the cumulative step size that corresponds to the highest.

### 5.1. Justification of Selected Features

In the following three sections we will explain why we have selected the features indices corresponding to the highest accuracy cumulative feature index.

#### 5.1.1. UnderFitting

With reference to Figure 2, we have labelled area corresponding to the low accuracy region as “biased”. The reason we have called it a biased region is that as in the initial cumulative steps, we have only a few features that cannot explain the complex relationship that exists between the features and the condition. A pattern can be observed in all the Table 2, Table 3, Table 4 and Table 5, whereas the number of selected features are added in the classifier, the 10-fold accuracy of the classifiers starts to increase.

#### 5.1.2. Over Fitting

With reference to Figure 2, we have labelled another area corresponding to the low accuracy region as “variance”. The reason we have called it a variance region is that after a large number of features are added for the classifier, the relationship between the features and the condition becomes too much complex and it could not well generalize on the new test data. A pattern can be observed in all the Table 6, Table 7, Table 8 and Table 9, whereas the number of selected features are increased in number, the 10-fold accuracy of the classifiers starts to decrease.

#### 5.1.3. Selected Features

We have selected the features based on the highest accuracy value as presented in the Figure 2 “Selected Features” marked area. Details of the cumulative feature step and the number of features corresponding to each of the classifiers are described in Table 10. For specific details of the selected indices on the three highest performing classifiers, Support Vector Machine, Logistic Classifier and Linear Discriminant Classifier, please see Tables S6–S8 in the Supplementary Materials section.

### 5.2. Combined Dataset Accuracy Using 10-Fold Cross Validation

A comparison of our Stage feature selection-based classifiers with the state of the art methods is presented in Table 11. Where first the correlation-based connectivity matrix is arranged on all the sites, ignoring the sites intrinsic variability, then a 10-fold cross-validation-based accuracy, sensitivity and specificity is given in the mentioned table. Our 3-Stage-based feature selection results outperformed state of the art results. Specifically, Logistic classifier accuracy and sensitivity is the highest when compared to the rest of classifiers, similarly, the highest specificity is observed in the SVM classifier. Hence, based on all the presented results, Improved performance based on all the three accuracy metrics is observed using our proposed feature selection approach which proves the effectiveness and robustness of our approach.

### 5.3. Site Wise Accuracy Using 5-Fold Cross Validation

In this experiment, we have demonstrated the discriminating power of selected features on the individual sites using the 5-fold accuracy metric. We compared our site-wise accuracy results with the state of the art works in the literature. Our experiment validates the robustness of the selected features that we selected using the 3-Stage baed approach on the combined dataset experiment as mentioned in the previous section. We have presented the 5-fold accuracy, sensitivity and specificity of various classifiers that are shown in Table 12. A reason for choosing the 5-fold accuracy metric is consistent with the results shown by previous works on the same dataset. Our 3-Stage Features-based classifiers have shown improved accuracy in 13 of the 17 sites, specifically as with the combined sites data results, here again, logistic classifier has outperformed in the 5-fold accuracy comparison with the state of the art methods in 13 out of the 17 sites.

## 6. Discussion

In this section, we will explain the usefulness and importance of the learned discriminant features that we obtained using the Teacher-Student neural network-based feature selection approach. It is pertinent to mention here that our features are not specific to autistic or healthy subjects. In fact, these features are related to both the autistic and healthy control subjects, hence they are referred to as altered or discriminating features related to both the condition. The selected discriminating features are the subset of the total 4005 pair-wise connected regions, hence any feature is the pair in the lookup of 4005 which underscores that for each feature we get the connected region from the 90×90 connectivity matrix.

### 6.1. Connectogram for the Brain Region Network

**Figure 3:**
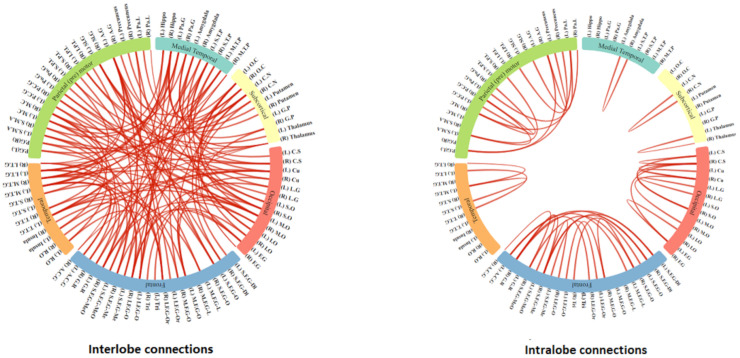
Brain’s lobes connectogram at the interlobe and intralobe level.

We have chosen a set of 154 features as these features are common among the three highest performing classifiers that are in, Support Vector Machine, Logistic Classifier and the Linear Discriminant classifier. Each of the 4005 sets of pair-wise connected regions correspond to two regions in the original 90×90 connectivity matrix, therefore, for each feature we have set a value of 1 where the regions are present and 0 elsewhere to display these discriminating features in the two connectograms corresponding to interlobe and intralobe connections in Figure 3.

#### 6.1.1. Connectivity in the Intralobe Network

Underconnectivity in the intralobe connections is visible as shown in the Figure 3 lobe features connectogram with most affected areas correspond to Temporal, Medial-Temporal and Subcorital lobes. Medial Temporal lobe has many key functionalities in the human life cycle, most importantly this is linked to the functionality of a person cognitive skills and attention that is required to perform a task [57]. Altered connectivity pattern in this part of the brain lobe and other lobes as mentioned above have also been found to be affecting the person who has autism as shown in the works of [58,59,60,61].

#### 6.1.2. Connectivity in the Interlobe Network

Altered connectivity patterns are visible in the brain Frontal and Parietal lobes in the interlobe connection network as shown in Figure 3 intralobe section on the selected features. The frontal lobe is the largest part of the brain that consists of two-third of the brain region has a variety of functionalities related to person’s mood, personality, cognition and social behaviour [62], whereas, the parietal lobe of the brain processes sensory information from the body and processing of arithmetic information, which are the key functions that are performed in this part of the brain [63].

**Figure 4:**
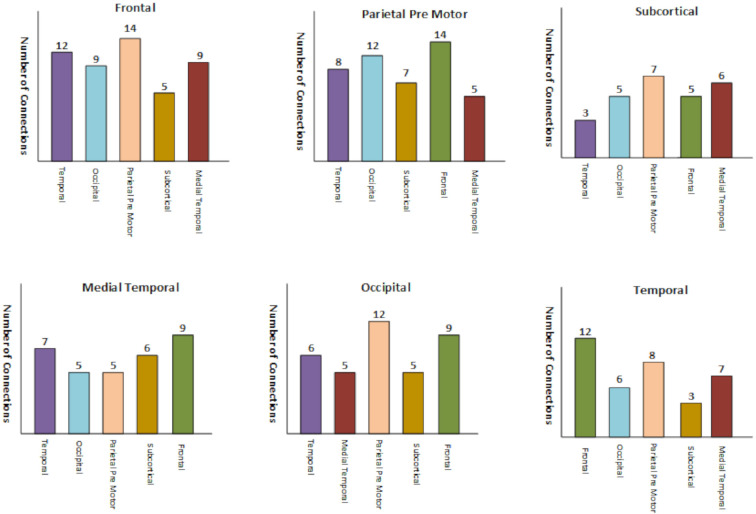
Brain’s Lobe wise connection statistics comparison level.

Due to denseness of the brain interlobe connectogram, we have shown the brain lobe wise count statistics in Figure 4 where altered connectivity counts are displayed lobe wise in the interconnection network data of the lobes. These altered connectivity pattern in the mentioned lobes are also consistent with the previous works [64,65,66,67].

### 6.2. Alterations in Brain’s Hemisphere Connectivity Patterns

**Figure 5:**
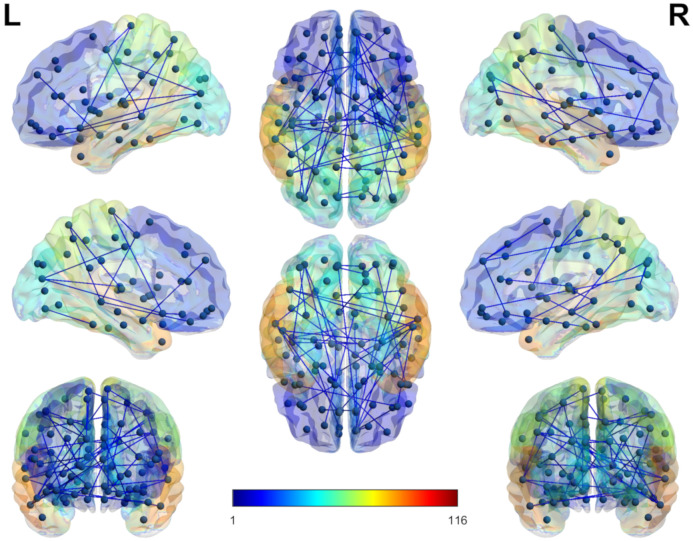
Brain node Connectivity network in **Left**, **Middle** and **Right** areas Using BrainNet-viewer tool.

The brain is perceived as a pair of two hemispheres consisting of a left hemisphere and a right hemisphere that form two interconnected units of the brain which are thought to have a key role in the functioning of the normal brain [68]. Set of node connections based on brain parcellation template with the inter and intra connections between the two hemispheres reveal patterns that are of key importance in many brain disorders such as ADHD [69], Alzheimer [70], Parkinson [71] and Schizophrenia [72] disorders. ASD has also proved to be exhibiting the altered connectivity patter in the brain two hemispheres as demonstrated in the works of [73,74]. To prove the altered connectivity patterns in the autistic and healthy control subjects we have used the famous tool developed by [75]. For this purpose we have selected high performing classification features that showed better results in the three mentioned classifiers as described int the feature selection section. BrainNet-viewer tool requires the set of nodes and the connected edges between those nodes, where as in our case we have set of discrimination features. For this purpose first we converted those features to the node and edge set using the 4005 lookup vector, where the node pair corresponds to feature index and for edge we set the value to 1.We have shown brain areas connectivity regions from three sides, that is left, middle and right are shown in Figure 5 using the BrainNet-viewer tool. It can be observed that the inter connectivity is higher in the brain hemisphere as opposed to the number of connection in each of the hemispheres. For better visualization using the Brain net viewer, we have changed the four critical parameters of the tool, that is the colour of each node was made thicker, node size was set to the fixed unit, weight corresponds to each of the edges was set to a fixed value and lastly, the opacity value of the surface was made lighter so that nodes and edges got more visible. Although all the nodes correspond to the selected features can be used to display on the BrainNet-viewer, but this will lead to clutter and a lack of clarity. To avoid this problem, only the top 50 features are used to select the nodes and connectivity between those nodes so that a better visualization could be achieved.

**Figure 6:**
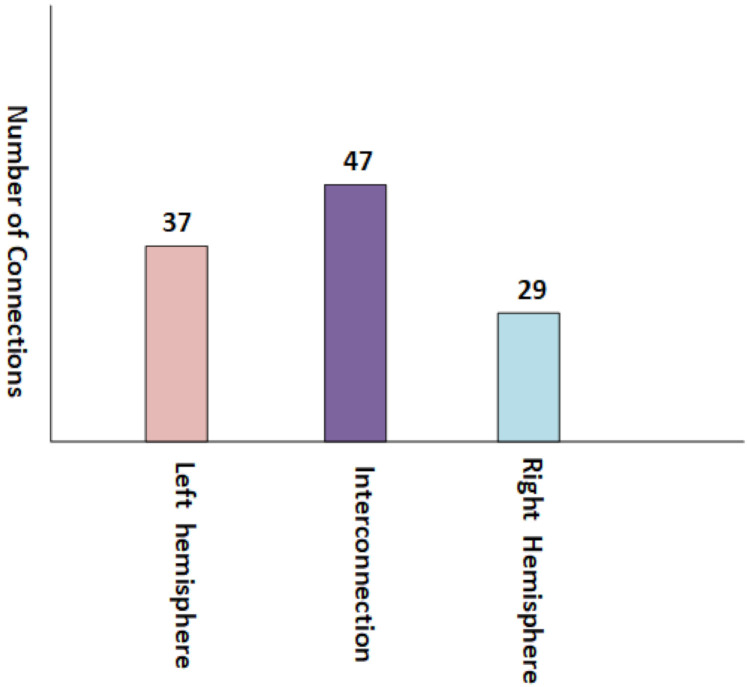
Brain’s nodes connection network counts between inter hemisphere and Intra hemisphere.

For better visualization, we have presented the connection counts between each hemisphere and in a hemisphere in Figure 6. An altered connectivity count is evident between the brain hemisphere, which is consistent with the work found in [76].

## 7. Conclusions

In this study, we have proposed a Teacher Student neural network-based feature selection approach to get the most discriminant features on the ABIDE preprocessed dataset. We have demonstrated the usefulness and importance of our features using various classifiers and compared our results with the state of the art methods at overall and site-wise level. We also have discussed the significance of our features using the brain anatomical analysis at the interlobe, intralobe and hemispherical level. We believe that based on our highly accurate results and detailed discussion, our methodology can be deployed in the clinical domain after discussion with the relevant experts and after checking the robustness of our methodology on more datasets on the autism disorder. In future studies, we will experiment to make this feature selection a part of an End-To-End deep learning model so that selection of features and classifiers are both packaged in one unified framework.

## Figures and Tables

**Table 1:** Autism Brain Imaging Data Exchange (ABIDE) Preprocessed Data from all sites.

		Participants	
Sr No.	Site Name	Autistic Subjects	Healthy Controls
1	Caltech	19	18
2	CMU	14	13
3	KKI	20	28
4	Leuven	29	34
5	MaxMun	24	28
6	NYU	75	100
7	OHSU	12	14
8	OLIN	19	15
9	PITT	29	27
10	SBL	15	15
11	SDSU	14	22
12	Stanford	19	20
13	Trinity	22	25
14	UCLA	54	44
15	UM	66	74
16	USM	46	25
17	Yale	28	28
**Total**		**505**	**530**
		**1035**	

**Table 2:** 10-Fold Results based on the Top 10 Selected Features based on Proposed 3-Stage Approach.

Sr No.	Classifier Name	Accuracy	Sensitivity	Specificity
1	LR	0.51	0.56	0.6
2	SVM	0.45	0.4	0.36
3	LD	0.49	0.51	0.54
4	RF	0.54	0.48	0.51
5	DT	0.31	0.34	0.29

**Table 3:** 10-Fold Results based on the Top 30 Selected Features based on Proposed 3-Stage Approach.

Sr No.	Classifier Name	Accuracy	Sensitivity	Specificity
1	LR	0.62	0.59	0.63
2	SVM	0.53	0.59	0.62
3	LD	0.51	0.58	0.6
4	RF	0.56	0.49	0.48
5	DT	0.5	0.49	0.5

**Table 4:** 10-Fold Results based on the Top 60 Selected Features based on Proposed 3-Stage Approach.

Sr No.	Classifier Name	Accuracy	Sensitivity	Specificity
1	LR	0.68	0.63	0.58
2	SVM	0.65	0.68	0.7
3	LD	0.7	0.61	0.62
4	RF	0.61	0.63	0.58
5	DT	0.59	0.51	0.6

**Table 5:** 10-Fold Results based on the Top 90 Selected Features based on Proposed 3-Stage Approach.

Sr No.	Classifier Name	Accuracy	Sensitivity	Specificity
1	LR	0.73	0.58	0.65
2	SVM	0.75	0.65	0.68
3	LD	0.73	0.71	0.69
4	RF	0.69	0.53	0.46
5	DT	0.63	0.51	0.8

**Table 6:** 10-Fold Results based on the Top 500 Selected Features based on Proposed 3-Stage Approach.

Sr No.	Classifier Name	Accuracy	Sensitivity	Specificity
1	LR	0.78	0.69	0.7
2	SVM	0.79	0.75	0.74
3	LD	0.74	0.7	0.67
4	RF	0.65	0.51	0.59
5	DT	0.62	0.58	0.61

**Table 7:** 10-Fold Results based on the Top 1000 Selected Features based on Proposed 3-Stage Approach.

Sr No.	Classifier Name	Accuracy	Sensitivity	Specificity
1	LR	0.73	0.65	0.74
2	SVM	0.74	0.71	0.72
3	LD	0.7	0.69	0.68
4	RF	0.61	0.6	0.63
5	DT	0.6	0.61	0.5

**Table 8:** 10-Fold Results based on the Top 1500 Selected Features based on Proposed 3-Stage Approach.

Sr No.	Classifier Name	Accuracy	Sensitivity	Specificity
1	LR	0.6	0.61	0.65
2	SVM	0.7	0.69	0.73
3	LD	0.62	0.64	0.61
4	RF	0.5	0.56	0.59
5	DT	0.36	0.39	0.4

**Table 9:** 10-Fold Results based on the Top 2000 Selected Features based on Proposed 3-Stage Approach.

Sr No.	Classifier Name	Accuracy	Sensitivity	Specificity
1	LR	0.51	0.55	0.52
2	SVM	0.51	0.55	0.52
3	LD	0.5	0.48	0.51
4	RF	0.31	0.29	0.33
5	DT	0.33	0.32	0.3

**Table 10:** Classifiers’ cumulative feature selection step and length.

Sr No.	Classifier Name	Max Cumulative Feature Step	Features Count
**1**	LR	5	256
**2**	RF	2	103
**3**	DT	1	52
**4**	SVM	3	154
**5**	LD	3	154

**Table 11:** 10-Fold Results Comparison of our 3-Stage approach-based selected Features on an all-Site combined dataset.

Sr No.	Classifier Name	Accuracy	Sensitivity	Specificity
1	LR (Ours)	**0.82**	**0.83**	**0.84**
2	LD (Ours)	**0.82**	**0.83**	**0.88**
3	SVM (Ours)	**0.81**	**0.80**	**0.92**
4	RF (Ours)	0.70	0.67	0.80
5	DT (Ours)	0.57	0.60	0.64
6	Heinsfeld et al., 2018 [44]	0.63	0.58	0.67
7	Taban Eslami et al., 2019 [48]	0.67	0.63	0.71
8	Ke Niu et al., 2020 [50]	0.73	0.74	0.71
9	Zeinab et al., 2020 [51]	0.70	0.77	0.61

**Table 12:** Proposed 3-Stage selected features’ 5-Fold Accuracy Comparison on each individual Site dataset.

Sr	Site	SVM (Ours)	RF (Ours)	DT (Ours)	LR (Ours)	LD (Ours)	Heinsfeld et al., 2018 [44]	Taban Eslami et al., 2019 [48]	Ke Niu et al., 2020 [50]	Zeinab et al., 2020 (5 fold) [51]
1	**Caltech**	**0.83**	0.65	0.53	0.78	0.67	0.52	0.52	0.66	0.54
2	**CMU**	**0.84**	0.7	0.6	0.71	0.6	0.45	0.68	0.63	0.7
3	**KKI**	0.62	0.6	0.58	0.66	0.6	0.58	0.69		**0.72**
4	**Leuven**	0.66	0.55	0.63	0.63	**0.76**	0.51	0.61	0.62	0.65
5	**MaxMun**	0.59	0.49	0.48	**0.61**	0.47	0.54	0.48		0.46
6	**NYU**	**0.78**	0.69	0.6	**0.78**	0.64	0.64	0.68	0.7	0.65
7	**OHSU**	0.5	0.5	0.6	0.74	0.69	0.74	**0.82**		0.57
8	**Olin**	0.52	0.59	**0.7**	**0.7**	**0.7**	0.44	0.65		0.58
9	**Pitt**	0.75	0.69	0.51	**0.78**	0.72	0.59	0.67	0.69	0.69
10	**SBL**	**0.66**	0.63	0.56	**0.66**	0.59	0.46	0.51		0.56
11	**SDSU**	0.61	0.64	0.61	0.69	0.69	0.63	0.63	0.69	**0.75**
12	**Stanford**	0.69	0.69	0.66	**0.71**	0.58	0.48	0.64	0.61	0.48
13	**Trinity**	0.46	0.66	0.5	0.52	0.63	0.61	0.54	**0.69**	0.61
14	**UCLA**	0.69	0.69	0.46	**0.77**	0.66	0.57	0.73	0.75	0.69
15	**UM**	0.7	**0.71**	0.52	**0.71**	0.63	0.62	0.68	0.68	0.66
16	**USM**	0.71	0.76	0.76	**0.8**	0.74	0.57	0.63	0.8	0.77
17	**Yale**	**0.75**	0.6	0.49	**0.8**	0.61	0.53	0.63	0.69	0.69

## Supplementary Materials

The following are available at https://www.mdpi.com/2076-3425/10/10/754/s1. Table S1: Teacher Student Neural Network, Table S2: Teacher Neural Network Parameter Settings, Table S3: Student Neural Network, Table S4: Student Neural Network Parameter Settings, Table S5: Automated Anatomical Label (AAL) regions, Table S6: Support Vector Machine Features List indices in the 4005 lookup, Table S7: Logistic Classifier Features List indices in the 4005 lookup, Table S8: Linear Discriminant Features List indices in the 4005 lookup.

## Abbreviations

The following abbreviations are used in this manuscript:

ABIDE	Autism Brain Imaging Data Exchange
ASD	Autism Spectrum Disorder
DT	Decision Trees
LD	Linear Discriminant
rs-fMRI	Resting State Functional Magnetic Resonance Imaging
RF	Random Forests
SVM	Support Vector Machine
