# Supplementary material for: A Three-Stage Teacher, Student Neural Networks and Sequential Feed Forward Selection-Based Feature Selection Approach for the Classification of Autism Spectrum Disorder
Source: Brain Sci. 2020 Oct 19;10(10):754. doi: 10.3390/brainsci10100754 (PMC7603385; doi:10.3390/brainsci10100754)
Supplement: Supplementary file 1 [file brainsci-10-00754-s001.pdf]

Supplementary Materials

Table S1: Teacher Student Neural Network

Sr. No	Layer Name	Neurons	Activation
1	Input	4005	----
2	Dense_1	3000	tanh
3	Dropout_1	0.3	----
4	Dense_2	1500	tanh
5	Dropout_2	0.3	----
6	Dense_3	1000	tanh
7	Dropout_3	0.2	----
8	Dense_4	500	tanh
9	Dropout_4	0.1	----
10	Dense_5	100	tanh
11	Dropout_5	0.1	----
12	Dense_6	50	tanh
13	Dropout_6	0.1	----
14	Dense_7	10	tanh
15	Dropout_7	0.1	----
16	Dense_9	5	tanh
17	Output	1	sigmoid

Table S2: Teacher Neural Network Parameter Settings

**Parameter**	**Value**
Optimizer	"Adadelta"
Learning rate	0.001
Loss function	"binary_crossentropy"
Batch Size	16
Epochs	500

Table S3: Student Neural Network

**Sr. No**	**Layer Name**	**Neurons**	**Activation**
1	Input	4005	---
2	Hidden	100	"relu"
3	Output	5	"relu"

Table S4: Student Neural Network Parameter Settings

**Parameter**	**Value**
Optimizer	"Adadelta"
Learning rate	0.01
Loss function	"mse"
Batch Size	16
Epochs	1000

Table S5: **Automated Anatomical Label (AAL) regions**

Labels	Regions	Regions	abbr.
1	Precentral_L	Precentral gyrus	PreCG.L
2	Precentral_R	Precentral gyrus	PreCG.R
3	Frontal_Sup_L	Superior frontal gyrus, dorsolateral	SFGdor.L
4	Frontal_Sup_R	Superior frontal gyrus, dorsolateral	SFGdor.R
5	Frontal_Sup_Orb_L	Superior frontal gyrus, orbital part	ORBsup.L
6	Frontal_Sup_Orb_R	Superior frontal gyrus, orbital part	ORBsup.R
7	Frontal_Mid_L	Middle frontal gyrus	MFG.L
8	Frontal_Mid_R	Middle frontal gyrus	MFG.R
9	Frontal_Mid_Orb_L	Middle frontal gyrus, orbital part	ORBmid.L
10	Frontal_Mid_Orb_R	Middle frontal gyrus, orbital part	ORBmid.R
11	Frontal_Inf_Oper_L	Inferior frontal gyrus, opercular part	IFGoperc.L
12	Frontal_Inf_Oper_R	Inferior frontal gyrus, opercular part	IFGoperc.R
13	Frontal_Inf_Tri_L	Inferior frontal gyrus, triangular part	IFGtriang.L
14	Frontal_Inf_Tri_R	Inferior frontal gyrus, triangular part	IFGtriang.R
15	Frontal_Inf_Orb_L	Inferior frontal gyrus, orbital part	ORBinf.L
16	Frontal_Inf_Orb_R	Inferior frontal gyrus, orbital part	ORBinf.R
17	Rolandic_Oper_L	Rolandic operculum	ROL.L
18	Rolandic_Oper_R	Rolandic operculum	ROL.R
19	Supp_Motor_Area_L	Supplementary motor area	SMA.L
20	Supp_Motor_Area_R	Supplementary motor area	SMA.R
21	Olfactory_L	Olfactory cortex	OLF.L
22	Olfactory_R	Olfactory cortex	OLF.R
23	Frontal_Sup_Medial_L	Superior frontal gyrus, medial	SFGmed.L
24	Frontal_Sup_Medial_R	Superior frontal gyrus, medial	SFGmed.R
25	Frontal_Mid_Orb_L	Superior frontal gyrus, medial orbital	ORBsupmed.L
26	Frontal_Mid_Orb_R	Superior frontal gyrus, medial orbital	ORBsupmed.R
27	Rectus_L	Gyrus rectus	REC.L
28	Rectus_R	Gyrus rectus	REC.R
29	Insula_L	Insula	INS.L

30	Insula_R	Insula	INS.R
31	Cingulum_Ant_L	Anterior cingulate and paracingulate gyri	ACG.L
32	Cingulum_Ant_R	Anterior cingulate and paracingulate gyri	ACG.R
33	Cingulum_Mid_L	Median cingulate and paracingulate gyri	DCG.L
34	Cingulum_Mid_R	Median cingulate and paracingulate gyri	DCG.R
35	Cingulum_Post_L	Posterior cingulate gyrus	PCG.L
36	Cingulum_Post_R	Posterior cingulate gyrus	PCG.R
37	Hippocampus_L	Hippocampus	HIP.L
38	Hippocampus_R	Hippocampus	HIP.R
39	ParaHippocampal_L	Parahippocampal gyrus	PHG.L
40	ParaHippocampal_R	Parahippocampal gyrus	PHG.R
41	Amygdala_L	Amygdala	AMYG.L
42	Amygdala_R	Amygdala	AMYG.R
43	Calcarine_L	Calcarine fissure and surrounding cortex	CAL.L
44	Calcarine_R	Calcarine fissure and surrounding cortex	CAL.R
45	Cuneus_L	Cuneus	CUN.L
46	Cuneus_R	Cuneus	CUN.R
47	Lingual_L	Lingual gyrus	LING.L
48	Lingual_R	Lingual gyrus	LING.R
49	Occipital_Sup_L	Superior occipital gyrus	SOG.L
50	Occipital_Sup_R	Superior occipital gyrus	SOG.R
51	Occipital_Mid_L	Middle occipital gyrus	MOG.L
52	Occipital_Mid_R	Middle occipital gyrus	MOG.R
53	Occipital_Inf_L	Inferior occipital gyrus	IOG.L
54	Occipital_Inf_R	Inferior occipital gyrus	IOG.R
55	Fusiform_L	Fusiform gyrus	FFG.L
56	Fusiform_R	Fusiform gyrus	FFG.R
57	Postcentral_L	Postcentral gyrus	PoCG.L
58	Postcentral_R	Postcentral gyrus	PoCG.R
59	Parietal_Sup_L	Superior parietal gyrus	SPG.L
60	Parietal_Sup_R	Superior parietal gyrus	SPG.R
61	Parietal_Inf_L	Inferior parietal, but supramarginal and angular gyri	IPL.L
62	Parietal_Inf_R	Inferior parietal, but supramarginal and angular gyri	IPL.R
63	SupraMarginal_L	Supramarginal gyrus	SMG.L
64	SupraMarginal_R	Supramarginal gyrus	SMG.R
65	Angular_L	Angular gyrus	ANG.L
66	Angular_R	Angular gyrus	ANG.R
67	Precuneus_L	Precuneus	PCUN.L
68	Precuneus_R	Precuneus	PCUN.R
69	Paracentral_Lobule_L	Paracentral lobule	PCL.L

70	Paracentral_Lobule_R	Paracentral lobule	PCL.R
71	Caudate_L	Caudate nucleus	CAU.L
72	Caudate_R	Caudate nucleus	CAU.R
73	Putamen_L	Lenticular nucleus, putamen	PUT.L
74	Putamen_R	Lenticular nucleus, putamen	PUT.R
75	Pallidum_L	Lenticular nucleus, pallidum	PAL.L
76	Pallidum_R	Lenticular nucleus, pallidum	PAL.R
77	Thalamus_L	Thalamus	THA.L
78	Thalamus_R	Thalamus	THA.R
79	Heschl_L	Heschl gyrus	HES.L
80	Heschl_R	Heschl gyrus	HES.R
81	Temporal_Sup_L	Superior temporal gyrus	STG.L
82	Temporal_Sup_R	Superior temporal gyrus	STG.R
83	Temporal_Pole_Sup_L	Temporal pole: superior temporal gyrus	TPOsup.L
84	Temporal_Pole_Sup_R	Temporal pole: superior temporal gyrus	TPOsup.R
85	Temporal_Mid_L	Middle temporal gyrus	MTG.L
86	Temporal_Mid_R	Middle temporal gyrus	MTG.R
87	Temporal_Pole_Mid_L	Temporal pole: middle temporal gyrus	TPOmid.L
88	Temporal_Pole_Mid_R	Temporal pole: middle temporal gyrus	TPOmid.R
89	Temporal_Inf_L	Inferior temporal gyrus	ITG.L
90	Temporal_Inf_R	Inferior temporal gyrus	ITG.R

Table S6 : Support Vector Machine Features List indices in the 4005 lookup

Sr No.	Indices
1	3604
2	3534
3	2925
4	3705
5	839
6	1950
7	2369
8	2046
9	3934
10	2674
11	2970
12	283
13	2341
14	3700
15	2293
16	67
17	2654
18	83
19	3218
20	2306
21	380
22	3927
23	158
24	2677
25	3464
26	1160
27	3252
28	454
29	2444
30	3456
31	201
32	2929
33	2877
34	3303
35	1237
36	1744
37	3429

38	1523
39	1831
40	1296
41	1637
42	1619
43	3526
44	1303
45	3704
46	1519
47	3582
48	976
49	2432
50	2477
51	3157
52	2767
53	1260
54	3658
55	1538
56	2728
57	945
58	1465
59	3331
60	2398
61	3010
62	2527
63	928
64	2864
65	1862
66	3815
67	2765
68	3992
69	745
70	3827
71	2465
72	3942
73	197
74	290
75	3059
76	2425
77	2411
78	3636
79	1797
80	2991
81	2780
82	3914

83	3255
84	2916
85	3946
86	1930
87	571
88	324
89	281
90	590
91	1473
92	3701
93	729
94	407
95	542
96	1674
97	358
98	3191
99	1243
100	1871
101	2289
102	1224
103	1976
104	1039
105	3301
106	939
107	1966
108	438
109	2883
110	769
111	1178
112	763
113	1305
114	3377
115	1725
116	412
117	1987
118	2119
119	3074
120	1622
121	3204
122	1267
123	2659
124	1114
125	2110
126	1177
127	98

128	1403
129	1617
130	1824
131	3424
132	2129
133	64
134	2049
135	2358
136	3958
137	795
138	720
139	2868
140	69
141	2791
142	856
143	2057
144	2934
145	3994
146	1489
147	3029
148	1768
149	3020
150	245
151	1922
152	2632
153	898
154	3963

Table S7: Logistic Classifier Features List indices in the 4005 lookup

Sr No.	Indices
1	3604
2	3534
3	2925
4	3705
5	839
6	1950
7	2369
8	3934
9	2046
10	2674
11	283
12	3700
13	2341
14	2293
15	67
16	83
17	2654
18	2306
19	3927
20	3218
21	380
22	158
23	2677
24	3464
25	1160
26	454
27	2444
28	2929
29	3252
30	201
31	3303
32	3456
33	2970
34	1523
35	1744
36	1831
37	1237

38	3429
39	1296
40	1303
41	1637
42	2432
43	3526
44	2477
45	1619
46	1519
47	3582
48	3704
49	976
50	2767
51	1465
52	2728
53	3658
54	3157
55	928
56	2398
57	945
58	3331
59	2527
60	1260
61	2877
62	3010
63	3815
64	1538
65	2765
66	1862
67	3636
68	197
69	3992
70	2864
71	3942
72	290
73	2465
74	3059
75	2425
76	3827
77	3255
78	745
79	2991
80	3701
81	1797
82	2411

83	2780
84	571
85	281
86	2916
87	407
88	324
89	1473
90	3946
91	1930
92	590
93	358
94	1674
95	542
96	729
97	2289
98	3191
99	1871
100	1243
101	1976
102	1178
103	1966
104	1039
105	939
106	1114
107	769
108	3301
109	3377
110	1725
111	1224
112	3914
113	2659
114	1987
115	2110
116	2129
117	763
118	1622
119	1177
120	64
121	1403
122	98
123	438
124	2883
125	2119
126	1305
127	3074

128	1267
129	3958
130	2868
131	412
132	2049
133	2057
134	1824
135	3204
136	795
137	2791
138	3424
139	2358
140	2460
141	3029
142	2934
143	69
144	1489
145	720
146	1922
147	3020
148	856
149	3994
150	1617
151	822
152	3774
153	898
154	2988
155	3520
156	3768
157	2454
158	585
159	3963
160	2189
161	1951
162	3295
163	2632
164	3193
165	245
166	36
167	1719
168	2880
169	3351
170	761
171	3820
172	1239

173	415
174	828
175	1990
176	483
177	105
178	3798
179	206
180	1022
181	2224
182	3174
183	1141
184	3383
185	2270
186	2241
187	1078
188	1768
189	3409
190	1498
191	2081
192	3094
193	3702
194	3513
195	1834
196	3399
197	2563
198	2956
199	46
200	1216
201	195
202	1676
203	2047
204	2417
205	2087
206	51
207	1927
208	2526
209	118
210	3373
211	326
212	981
213	1948
214	1499
215	862
216	244
217	2824

218	3971
219	547
220	187
221	2145
222	134
223	2675
224	3746
225	1072
226	1152
227	783
228	3619
229	217
230	1518
231	759
232	967
233	2804
234	1597
235	1008
236	3107
237	168
238	1532
239	3689
240	2913
241	62
242	3038
243	2147
244	1517
245	754
246	3659
247	2099
248	1369
249	791
250	3797
251	3968
252	1179
253	3622
254	3587
255	381
256	409

Table S8 : Linear Discriminant Features List indices in the 4005 lookup

Sr No.	Indices
1	3604
2	3534
3	2925
4	3705
5	839
6	1950
7	2369
8	3934
9	2046
10	2674
11	283
12	3700
13	2341
14	2293
15	67
16	83
17	2654
18	2306
19	3927
20	3218
21	380
22	158
23	2677
24	3464
25	1160
26	454
27	2444
28	2929
29	3252
30	201
31	3303
32	3456
33	2970
34	1523
35	1744
36	1831
37	1237
38	3429

39	1296
40	1303
41	1637
42	2432
43	3526
44	2477
45	1619
46	1519
47	3582
48	3704
49	976
50	2767
51	1465
52	2728
53	3658
54	3157
55	928
56	2398
57	945
58	3331
59	2527
60	1260
61	2877
62	3010
63	3815
64	1538
65	2765
66	1862
67	3636
68	197
69	3992
70	2864
71	3942
72	290
73	2465
74	3059
75	2425
76	3827
77	3255
78	745
79	2991
80	3701
81	1797
82	2411
83	2780

84	571
85	281
86	2916
87	407
88	324
89	1473
90	3946
91	1930
92	590
93	358
94	1674
95	542
96	729
97	2289
98	3191
99	1871
100	1243
101	1976
102	1178
103	1966
104	1039
105	939
106	1114
107	769
108	3301
109	3377
110	1725
111	1224
112	3914
113	2659
114	1987
115	2110
116	2129
117	763
118	1622
119	1177
120	64
121	1403
122	98
123	438
124	2883
125	2119
126	1305
127	3074
128	1267

129	3958
130	2868
131	412
132	2049
133	2057
134	1824
135	3204
136	795
137	2791
138	3424
139	2358
140	2460
141	3029
142	2934
143	69
144	1489
145	720
146	1922
147	3020
148	856
149	3994
150	1617
151	822
152	3774
153	898
154	2988
